# Developing and validating five-construct model of customer satisfaction in beauty and cosmetic E-commerce

**DOI:** 10.1016/j.heliyon.2020.e04887

**Published:** 2020-09-14

**Authors:** Thuan Thi Nhu Nguyen

**Affiliations:** Tomas Bata University in Zlín, Czech Republic

**Keywords:** Customer satisfaction, Determinants, Beauty & cosmetic, Online shopping, Vietnam, Behavioral economics, International economics, Management, Marketing, Globalization

## Abstract

Grounded on the American Customer Satisfaction Framework (ACSF) of Bryant (1995), the European Customer Satisfaction Framework Model (ECSF) of Anderson and Fornell (2000), and Eleven-factor Customer Satisfaction Model of Hokanson (1995), this study develops reliable and valid five constructs of customer satisfaction theoretical model and build up a questionnaire in the unique context of Beauty and Cosmetic Online Shopping in the Vietnamese market. More specifically, we identified five main constructs including 5 implementation constructs (i.e., online shopping experience, customer service, external incentives, security/privacy, and personal characteristics), and one outcome construct (i.e., customer satisfaction). A detailed questionnaire was then developed with items for these constructs along with the questions on the personal characteristics of the respondents. The questionnaire was sent to randomly consumers via online channels including Facebook. The target participants are Vietnamese people who have experience in shopping online products like beauty and cosmetics. Based on 334 full responses have been received, we use Exploratory Factor Analysis (EFA) to test for 167 observations to ensure that all items in each scale reflected sufficiently the scope of each construct. This technique helps to identify the underlying dimensions of consumer satisfaction. Furthermore, we also employ Confirmatory Factor Analysis (CFA) to validate the constructs and items in questionnaire. To do this, we have used the sample of 167 responses. Finally, together with results from SEM models, our study contributes to providing a reliable and valid questionnaire which fully reflect for our self-constructed theoretical model of five constructs including Online shopping experience (OSE), External incentives (EI), Seller service (SS), and Security/privacy (SP), as well as personal characteristics (PC).

## 1 Introduction

“*Satisfaction influences repurchase intentions whereas dissatisfaction has been seen as a primary reason for customer defection or discontinuation of purchase*”.

- LaBarbera and Mazursky (1983) -

Customer Satisfaction (CS *hereafter*) is considered as a crucial factor which can affect the firm sales. In other words, CS exerts an influence on the foundation of any successful businesses including beauty and cosmetics in the extreme competitive market. This is possibly because of the fact that higher CS level plays a vital role in encouraging customers to repurchase their products or reuse services (Hoyer et al., 2001; Park et al., 2019). It can be understood as a measurement determining the happiness of customers regarding their consumed products or services. Indeed, it refers to the gap of customers' emotional response to their original expectations and that to the real values that they have received during both, purchasing and consumption periods. As such, following previous studies in the field (Rust and Oliver, 1993; Fornell, 1992; Oliver, 1999; Woodside et al., 1989; Kotler et al., 2000; Hansemark and Albinsson, 2004; Tam, 2004), we claim that CS reflects customers' attitudes based on their experience, thereby, it can be related to feelings of acceptance, happiness, relief, excitement, and delight (Hoyer et al., 2001). Prior literature has highlighted two fundamental concepts of CS: the first is the satisfaction of individuals for a specific transaction and the second is their overall (cumulative) satisfaction. The latter is previously proved to be most useful in assessing CS and customers’ post-purchase behaviours (Wang et al., 2004; Kuo et al., 2009; Deng et al., 2010).

In an ideal scenario that customers are satisfied with products/services, we expect that they might be willing to share their good experiences with social communities surrounding them particularly their friends and family members. Today, in this modern communication world, with a convenience of online channels via internet, their personal sharing has more significant impacts on people as more online users may be easily and quickly read those feedback. On the contrary, in a bad scenario that the customers are not fully satisfied, we anticipate that they may spread the news about their unfortunate or bad purchasing experience (Zairi, 2000). As such, the consequences that an online business will receive (the second case) tend to be significantly more severe because individuals, who are unsatisfied, may discontinue buying products or using the services, make complains, return the products, or even engage in negative word of mouth communication with their friend, family and particularly online communities. Despite the importance of building and retaining CS in strategic planning, it is challenging to know exactly why and how a customer is satisfied with a specific product.

Till date, several literatures have focused on this interesting and important aspects but the majority of them (see among others, Hallowell, 1996; Cronin et al., 2000; Sivadas and Baker Prewitt, 2000; Bowen and Chen, 2001; Shankar et al., 2003) placed on the role of CS as a vital construct in customer behaviour models and find that CS has significantly positive effects on the products repurchase and customer loyalty, and in turn, a corporate profitability. Businesses, therefore, may need to put their all efforts in improving CS levels by meeting customers' needs and wants. Needs refer to “*the felt deprivation of a customer*” while wants is “*the form taken by human needs as they are shaped by culture and individual personalit*y” (LaBarbera and Mazursky, 1983; Kotler et al., 2000). However, there exists a clear gap of literature which develops theoretical and empirical models related to determinants of CS for online businesses, and we also felt the need to develop and validate a questionnaire on CS in general and in the context of Vietnamese Beauty and Cosmetic Online Shopping (BSOS *hereafter*) in particular. We therefore fill this important gap and conduct our research in two separate studies. The *first* (this piece of work) is a research paper which developed and validated the Customer Satisfaction Survey (CSS) Scale in the BSOS with a Vietnamese sample. We expect to contribute this part to the methodology of CS literature. Typically, Nicholls (1998) has highlighted that the CSS can be one of the most effective approaches to assess key service elements in multiple service sectors and Gilbert et al. (2004) adopted Nicholls’ CSS and tested it across different cultures. In addition, Phau and Ferguson (2013) also validated CSS in fast food businesses with an Australian sample and they interestingly have found that customer (personal) service and service setting are among important dimensions of CS. The *second* study which is presented in a separate paper, will use the developed and validated questionnaire in the first one to conduct a survey for determinants of CS in BSOS in Vietnam and tested if the gender makes any differences. We believe that several managerial implications together with future directions for online businesses make our studies worthy to circulate.

The rationale to choose beauty and cosmetics as well as online shopping in Vietnam is three-fold. Firstly, it is undoubted that the popularity of the internet has enabled a wide range of online services, including online shopping businesses, in recent years in many countries including Vietnam – which is classified as an Asian, opening and developing economy. This is evident by a significant increase in the percentage of consumers who spent their time on shopping online. Secondly, the BSOS sector has widely assessed as one of the most active and healthiest markets in Vietnam, demonstrated by the growth rate of 115 per cent in 2016 relatively compared to the subsequent year. One can explain for this phenomenon through the rapid development of Vietnamese economy, along with more dynamic marketing activities as well as the enormous changes in business distributions. Giving an illustration for this point, we reveal that customers in this country can easily approach a variety of products and services via a number of online information channels for the benefits of suitable beauty and cosmetics. This is expected to result in an improvement of online businesses’ net sales within the BSOS sector.

More importantly, the changes in distribution method of Vietnamese businesses in recent years has continuously contributed to the higher demand for beauty and cosmetics in this country. For instance, there have been experiencing a dominance in online businesses penetration and social media trading since 2015. Consequently, when a customer likes to buy online products, their choices are very diversified as they are able to select any labelled or branded beauty and cosmetics products via online channels such as website or Facebook groups/pages. Even in rural areas across the country, customers are able to make any order and online businesses will deliver it to their door. This leads to a more convenience level in purchasing online stuffs than shopping in-store. Such distribution with great convenience and customers’ knowledge and awareness of the benefits of purchasing beauty and cosmetics via online channels have substantially promoted the market share of BSOS sector. Moreover, the recent development of Vietnamese economy (GDP growth rate of over 6.7% in 2016) and higher standard of living have helped to increase the purchasing power of Vietnamese consumers towards beauty and cosmetics products particularly and others generally. Last but not least, with the intention to mitigate the information gap and provide online Vietnamese businesses in BSOS industry with practical knowledge related to CS, we find a need to identify the CS constructs in this tremendously growing market, and develop an instrument for measuring these constructs and empirically validating the instrument using data from online shopping customers in this country. In term of literature, similar studies have constructed a questionnaire to either examine the gender differences in consumer behaviour for online financial transaction of cosmetics (Liu et al., 2013), or to explore the nexus of e-service quality and CS (Rita et al., 2019).

Furthermore, other research has placed on the developing and validating questionnaire in other areas and markets, for example, Bargas-Avila et al. (2010) has done this development and validation of a short questionnaire to estimate user satisfaction with e-Government portals; Huber et al. (2007) measured CS and customer value in services transactions, scale development, validation and cross-cultural comparison; Aletras et al. (2010) developed and preliminarily validated of a questionnaire to measure satisfaction with home care in Greece; Phau and Ferguson (2013) validated the CS survey scale in the fast food sector in Australia; and Boß et al. (2016) examined the reliability and validity of assessing user satisfaction with web-based health interventions. Unfortunately, to our best of knowledge, there is no studies identifying constructs for CS and proving a reliability and validity of the survey used to collect the data, in (i) beauty and cosmetics sector, (ii) online shopping; and (iii) Vietnamese context. We therefore aim at developing and validating a questionnaire on CS in the particular context of Beauty and Cosmetic Online Shopping in Vietnam.

## 2 Research background and developing customer satisfaction constructs

### 2.1 Online shopping experience (OSE) construct

The rapid evolution of technology has witnessed a shift either in a partial or a complete mode to the digital world, including shopping experience (Pantano and Priporas, 2016); therefore, OSE has been portrayed as a vital component in this global competition. Despite its importance, a body of literature has shown that understandings of OSE have still been nascent (Bilgihan et al., 2016; McLean and Wilson, 2016), particularly gender differences in consumer behaviour and decision making online.

Previous studies on gender differences have reported inconsistent findings. For example, men and women differ from each other in terms of their psychological behaviours when they attempt to make any purchases online (Wolin and Korgaonkar, 2003). In detail, men display more positive attitudes towards online shopping because they perceive the potential risks of purchasing online, familiarise with website design, and gain technology acceptance. However, Alreck and Settle (2002) state that women's attitudes towards online shopping are found to be similar with those of men even though it is obvious that women are more likely to show more positive attitudes towards shopping in general. Other studies have shown that women do not derive immense satisfaction in OSE compared with men (Doolin et al., 2005; Rodgers and Harris, 2003). From conflicting findings in previous studies, it is essential to devote more attention and seek better understandings of gender differences in OSE. This also stimulates the potential needs on the relationships among gender differences, OSE and cosmetics because the use of cosmetics is no longer exclusive to women (Liu et al., 2013).

### 2.2 Seller or customer service (SS) construct

As mentioned above, the BSOS sector is considered as an exponentially thriving yet intensively competitive business; therefore, the biggest challenge for online shopping is how to maintain adequate and efficient CS. To achieve this, it is essential for online businesses to bring the customers high-quality products and services that is argued to positively affect their loyalty (Gounaris et al., 2010). If SS is highly appreciated by the online shoppers, they will be likely to have favourable behavioural intentions (see Brady and Robertson, 2001). A demonstration for this is about website design quality, i.e., online shoppers can easily find all important and relevant information of the products including characteristics, functions, origin, and payments. Given that electronic services are considered as one of the most critical determinants for an online business’ success (Sharma and Lijuan, 2015). CS, therefore, will be positively affected by both, the attributes and characteristics of e-service quality through an excellent e-service quality (Blut et al., 2015). Importantly, delivery/fulfilment, which refers to activities of online businesses, is able to ensure that online shoppers could receive the correct order (i.e., delivery time and condition, order accuracy) (Blut et al., 2016). It is able to be assessed after the purchase and payment process was fully completed. Previous evidence has shown that compared to in-store shopping, it is more likely that customer post-payment dissonance occur during online shopping stages. This is simply because online shoppers could not see the purchased products/services in person before making a purchasing decision (Keng and Liao, 2013). Online businesses, consequently, need a guarantee and assurance for their customers that they will deliver correct products in an agreed time and date as well as other conditions. We therefore consider fulfilment as one of the determinants of e-service quality. Moreover, SS also include other aspects such as ordering, payment method, guarantee policy, and other customer services. If these elements are excellent, the CS might be increased as their purchasing experience is good.

### 2.3 External incentives (EI) construct

Previous studies have defined external incentive (EI) as a factor including several elements such as Price of products, Promotion activities and policy, Product attributes and quality, brands of products and source of opinion (Rita et al., 2019). These factors are expected to significantly affect customer experience and therefore CS. Typically, online shoppers can buy products and services with lower prices while they do not need to pay for other costs such as expenses of negotiating traffic, spending time, and using energy to compare product prices. This reduces the pressures for online shoppers resulting in comfortable and convenient feeling, which includes obtaining lower price and more accurate product information from different online sellers. In addition to this, the customers can obtain their favourable products and services more quickly than in-store shopping that may delay their purchasing (Yaylı and Bayram, 2012; Shin and Biocca, 2017). Furthermore, online businesses could offer better promotion policies such as reducing prices (EI), which in turn, affects CS. This is consistent with the study of Wang et al. (2011) who have argued that a customer is more likely to choose a retailer giving them more attractive promotion policy. This suggests that promotion and lower price plays vital roles in enhancing the consumers' perception of emotional value, and in turn, CS. Moreover, unique attributes and excellent quality of online beauty and cosmetics products also influence the customers’ trust and enjoyment, which leads to their higher satisfaction. Last but not least, brands of products may be bias customers that they offer them more value in terms of quality.

### 2.4 Security and privacy (SP) construct

Previous evidence (e.g. Liu et al., 2013; Chiang and Dholakia, 2003; Rita et al., 2019) has also highlighted the potential effects of security and privacy on CS. We therefore include this group of constructs into the survey and our theoretical model. More specifically, SP can be described as the security of credit card payments from customer purchasing transaction. In other words, it is considered as the privacy of shared information (Blut et al., 2016). In the situation that online businesses make their advertising activities and purchasing transactions via their website, they should increase the confidence of customers towards SP (e.g., security of personal information and payment details such as demographics, contact number, delivery address and credit card information) (Holloway and Beatty, 2008). Failure to do so may reduce the customers’ trust and as such their satisfaction can be destroyed. An effective website, according to Schmidt et al. (2008), is one which features well SP. It can protect online shoppers against fraud after they made a purchasing transaction. We argue that SP construct plays an important role in improving customer trust and CS.

### 2.5 Personal characteristics (PC)

We also include personal characteristics into the models and survey. According to Liebermann and Stashevsky (2002), PC comprising of customers’ gender, age, marital status, and education has significant impacts on their perceived risks of Internet usage. For example, Simon and Peppas (2005) has also noted that males and females reveal differential perceptions and satisfaction on the use of websites. As we consider e-commerce as one of the key facets of internet activity, we argue that the finding of Simon can be applicable to the context of online shopping. In addition, other factors (i.e., trust, commitment, relational embeddedness, and social interaction ties) have been also empirically examined by several prior research (Chang et al., 2015). The next category emphasizes transaction-related factors such as perceived price fairness, participation volume, Internet advertising, prior purchase experience, and satisfaction (e.g., Shiau and Chau, 2016; Zhang et al., 2015), where theories of transaction cost economics, expectation–confirmation, and social identity have been adopted.

### 2.6 Constructing the conceptual framework for determinants of CS in BCOS

**Figure 1:**
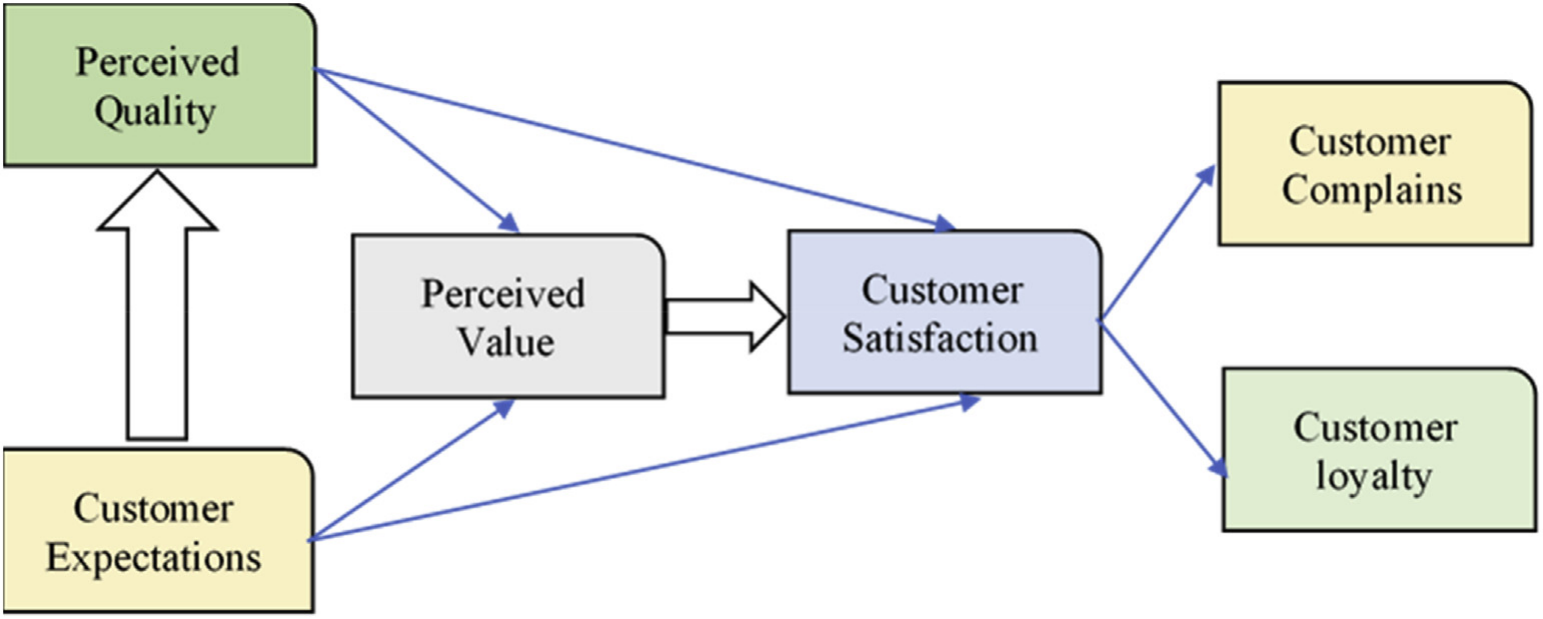
American customer satisfaction framework (ACSF).

Source: Bryant (1995).

**Figure 2:**
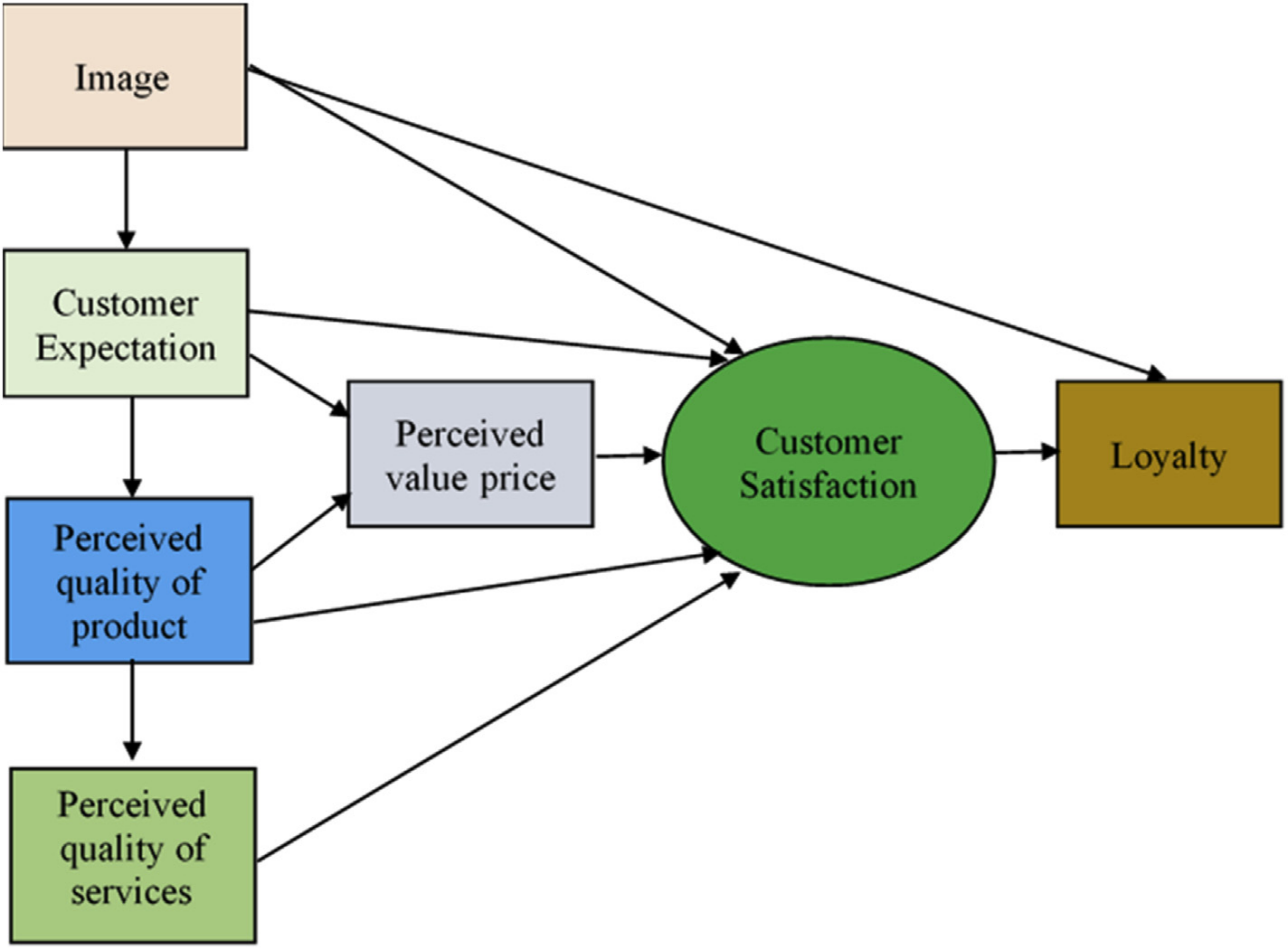
European customer satisfaction framework model (ECSF).

Source: Anderson and Fornell (2000)

**Figure 3:**
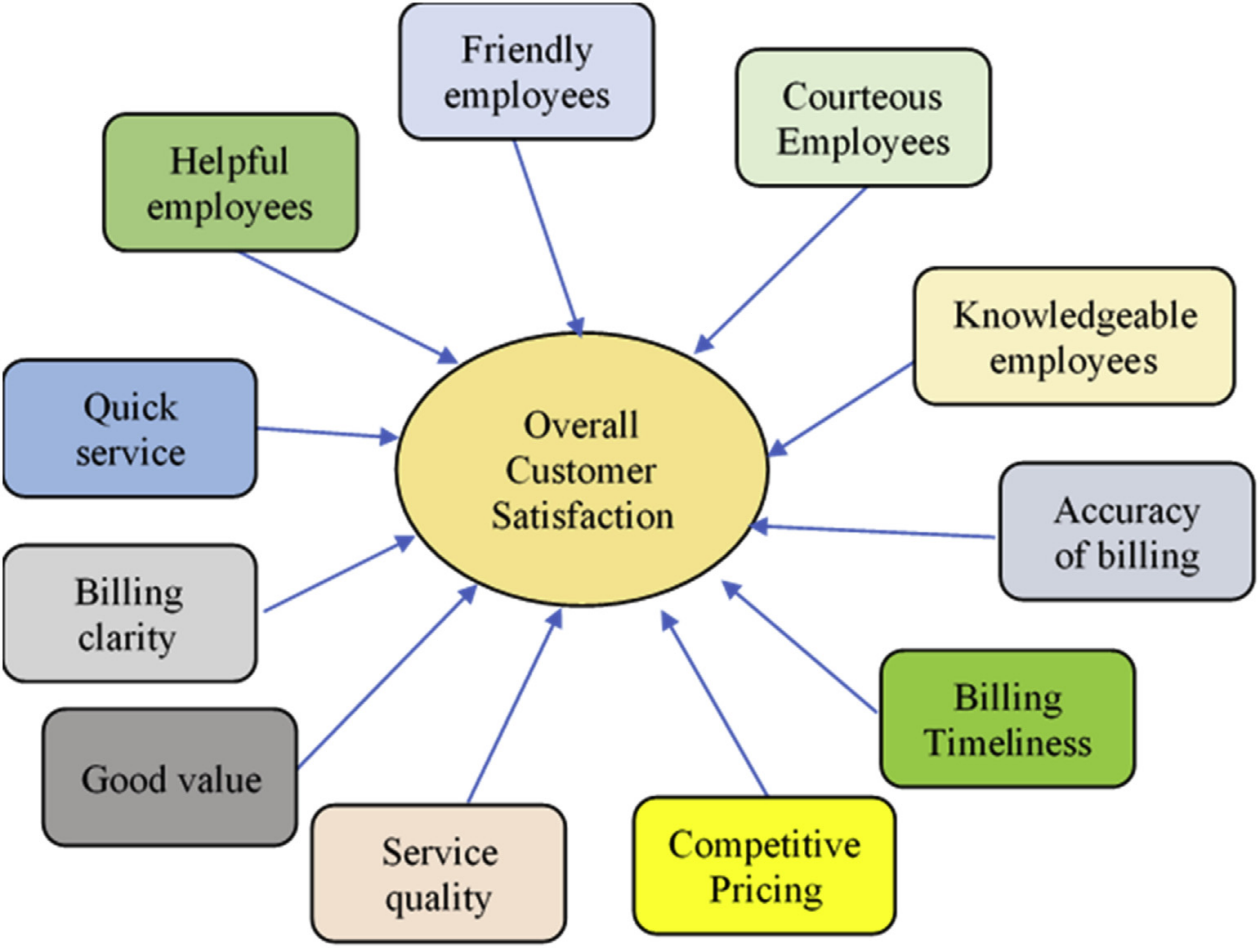
Factors that affect customer satisfaction.

Source: Hokanson (1995).

Previous studies have introduced several models of CS. While the American Customer Satisfaction Framework (ACSF) (Figure 1) has described the relationships between CS and its determinants such as customer expectation, perceived quality and perceived value (Bryant, 1995), the European Customer Satisfaction Framework Model (ECSF) (Figure 2) has added the image factor into the model and classifies perceived value of quality into the perceived quality of product and that of services, as well as of price (Anderson and Fornell, 2000). The former focuses on the customers' experience in physical stores and the latter focused on the re-purchase factors which affect CS. However, these factors are general, and it may not be fully applied to the unique context of Vietnam and BCOS. We have also been motivated by these ACSF and ECSF models by making a survey with a number of questions related to customers’ perception of price, quality and services. Furthermore, we consider 11 factors of model of Hokanson (1995) which consist of friendly staff, courteous staff, knowledgeable staff, helpful staff, the accuracy of payments, prompt payment, competitive prices, service quality, better value and payment of a clear and quick service (Figure 3). All of these theoretical determinants have been reflected in our self-constructed theoretical model with five-constructs.

**Figure 4:**
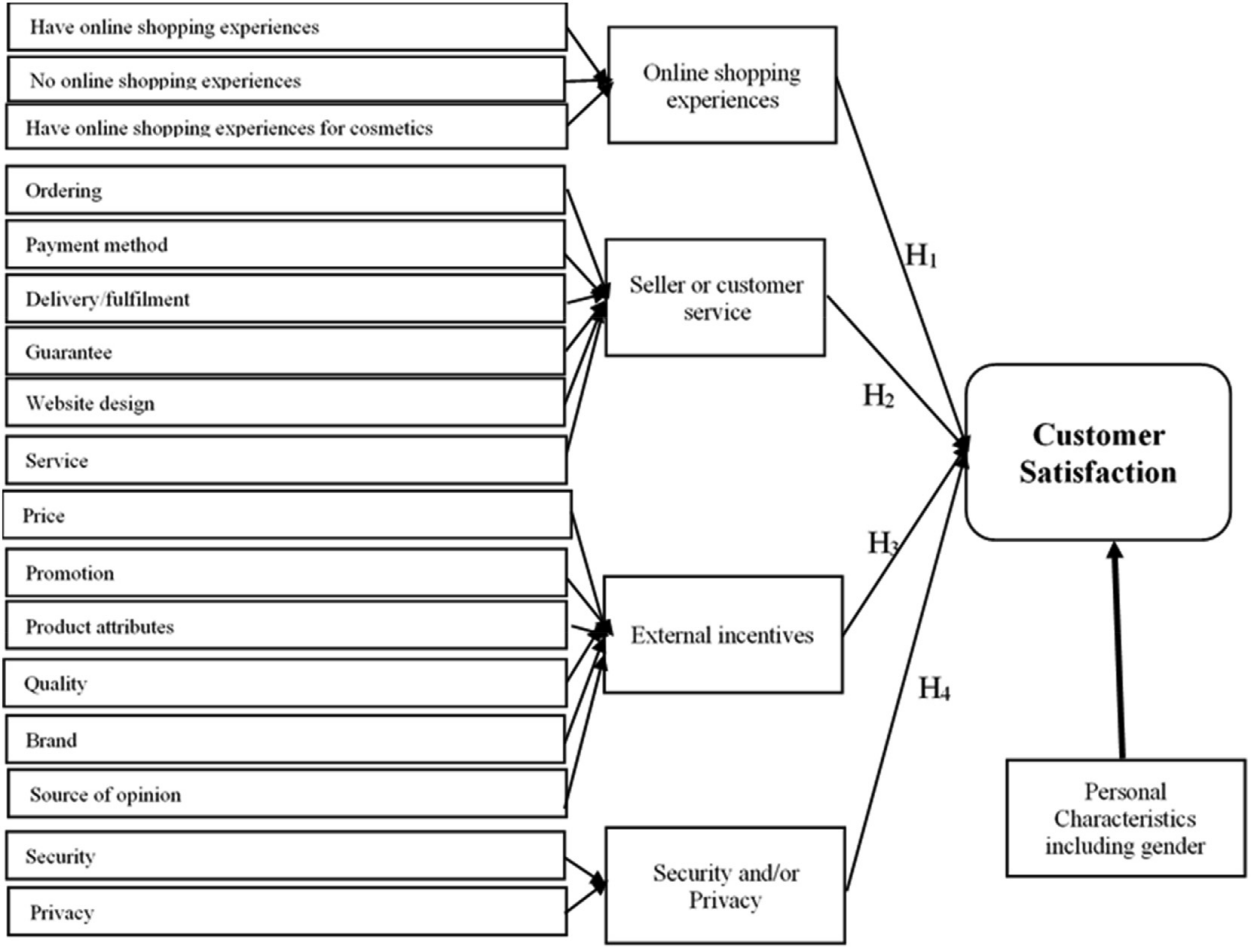
Five-construct theoretical model of customer satisfaction.

Source: Own research & Rita et al. (2019).

Furthermore, with the above five constructs (i.e., OSE; SS; EI; SP; PC) demonstrated in Section 2.1–2.5, we have constructed the below Figure 4 which shows a theoretical model. For the *first* construct (OSE), we classified the consumers into three types: (1) those who have OSE for all product, (2) those who have no OSE; and (3) those having OSE for beauty and cosmetics products. As mentioned earlier, we expect that customers' prior experience may affect their purchasing behaviour in the future and their judgement on new online products' orders may be compared to prior experience, and in turn, influence their CS. For the *second* construct (SS), it is likely to affect the CS because matters related ordering, payment method, delivery and fulfilment, guarantee, website design and customer service are imperative in creating great experience for online customers. If their trust on SS was reduced due to negative experience on these matters, their CS may be significantly and adversely affected. The *third* construct (EI) is built up upon on the traditional marketing mix which includes four P(s) (McCarthy, 1960): Product (i.e., Product attributes, Brand and Quality), Place, Promotion and Price. Place is ignored because of online shopping. We have already included the delivery in SS construct. Source of opinion is also added as an important element of EI. The last construct is SP. This has been added to the theoretical model of BCOS because SP is important to online shopping which is subject to the cybersecurity. For example, personal information and payment details of customers can be stolen by online businesses or internet hackers. For online businesses, it is no doubt that PS will affect CS. Compared to these previous frameworks, our model is constructed based on the situations and sectors’ characteristics, as such, it can fully reflect factors potentially influencing the CS in BCOS.

Consistent with previous CS studies, we have constructed our hypotheses as follows:

H1 There is a positive and significant relationship between online shopping experience and customer satisfaction

H2 There is a positive and significant relationship between seller service and customer satisfaction

H3 There is a positive and significant relationship between external incentives and customer satisfaction

H4 There is a positive and significant relationship between security and privacy and customer satisfaction

## 3 Research methodology and data collection procedure

This section explains the five-step development and validation of the questionnaire on CS. A wide range of reviews was undertaken in order to draft the initial items for the questionnaire. Subsequently, a two-round Delphi iterative consultation process (qualitative and quantitative) with a panel of experts (Keeney et al., 2006) was employed, which is widely used for the purpose of developing and validating a questionnaire (Fuentes-Blasco et al., 2010). Fourthly, a pilot study was conducted on a sample of 100 participants. Finally, a cross-validation was made through a main validation study including 334 participants.

Step one: initial development of the questionnaire

Perceived as the preliminary stage of Delphi research, the domain of interest, the formats and number of items were elucidated to develop the questionnaire following the review of relevant literature (Crocker and Algina, 1986). As mentioned in section 2, this research has identified one outcome construct (i.e. CS) and five main implementation constructs (i.e. OSE; EI; SS; and SP; and personal characteristics).

Our research initially conducted a review of existing literature related to (1) Consumer satisfaction and its determinants; (2) online shopping in different countries including Vietnam; (3) gender differences (if any). This stage is useful in finding the literature gap and identifying possible main factors affecting the consumer behavior. More specifically, we have carried out this step by using different databases from reliable sources including Google Scholar, Scopus, and Web of Science databases. The number of key words for searching are also used such as “customer satisfaction”, “determinants of customer satisfaction”, “online shopping”, “beauty and cosmetics industry”, “factors affecting customer satisfaction”, “Vietnam” and so on. We also combined these key words to obtain better searching results. For example, when we searched only the key word of “customer satisfaction” in Google Scholar, there was 2,420,000 results which are too large. Therefore, we have searched “customer satisfaction” and “online shopping”, the results have been significantly reduced to 271,000 searching results. We then continued combining these two key words with others to reduce the number of results while still relatively ensuring the quality of literature (i.e., relevant, reliable, etc.). Because we do not conduct a systematic literature review, hence, we have restricted our literature to studies closely relevant to our research questions, e.g., studies about factors affecting customer satisfaction, particularly in Vietnam and in beauty and cosmetic sector. Through a comprehensive analysis of the previous studies, we can propose a conceptual model (Figure 4) that combine the groups of factors and variables, and investigate their effects on CS. These constructs are selected because they are proved to be significantly related to CS, especially in the similar sectors and countries (see Bryant, 1995; Hokanson, 1995; Anderson and Fornell, 2000; Liu et al., 2013; Rita et al., 2019). In addition, we chose these constructs because they are supported theoretically and logically in the context of online shopping and in Vietnam.

The detailed concept and elements of each group of variables are indicated in Table 1 below.

Step two: qualitative Delphi study

**Table 1:** The concept of variables constructs.

Variables	Construct	Source
**Outcome construct**		
Consumer Satisfaction (CS)	Describe the satisfaction of consumers in buying online products	Bryant (1995); Hokanson (1995); Anderson and Fornell (2000); Liu et al. (2013); Rita et al. (2019), and so on.
**Implementation construct**		
Online Shopping Experience (OSE)	Describe consumers' prior experience in buying online products	Bryant (1995); Hokanson (1995); Anderson and Fornell (2000); Liu et al. (2013), Rita et al. (2019), and so on.
External Incentives (EI)	Describe incentives from the online businesses for consumers	
Customer Service (CS)	Describe the quality of online business services	
Security/Privacy (SP)	Describe the security and privacy of consumers when buying online products	
Personal Characteristics (PC)	Describe the demographics of the consumers and their frequency of internet use	

Individuals participating in a Delphi study must be recognised as acquiring substantial levels of appropriate expertise in the area being investigated (Davis, 1997). Thus, I compiled a list of 8 lecturers teaching Management- and Marketing-related modules at two universities in Southern Vietnam. By the time joining this step, these 8 lecturers were teaching some modules such as marketing research, retail management, digital marketing, consumer behaviour, or marketing strategy. In addition, they were active in conducting research in these two fields, with relevant experiences in publishing academic papers and presenting research at some national and international academic conferences. Therefore, their constructive feedback on the draft of this questionnaire, at this stage, would be useful for correcting any misinterpretations of the items. This stage aimed to avoid any confusion in designing the questionnaire survey, which helped to identify any sensitive and inappropriate information. In other words, it was beneficial in terms of making thorough evaluations for the accuracy of the questionnaire and its representativeness for corresponding constructs. These 8 experts were asked to circle any words or phrases in the draft of questionnaire items in which they were confused, then reword the statements to make those items more understandable, and finally make other constructive comments about the statements. Following the feedback from these 8 lecturers, there was no contradictions found in the appropriateness and sensitivity of the items in the questionnaire. The revisions they suggested were mostly focused on word choice. This is because the target population of the questionnaire was Vietnamese people having interests or experiences in shopping online products like beauty and cosmetics. Therefore, the choice of words and phrases in each item must be simple and easy for expressing the meaning and intent of the question. It must ensure that all respondents would make the same interpretations when they were filling in the questionnaire subsequently.

Step three: quantitative Delphi study

Based on the revised questionnaire items through qualitative Delphi study, another group of 15 experienced Vietnamese lecturers and doctoral researchers were invited to provide further ratings regarding the relevance of the revised items to the CS questionnaire. These 15 participants were teaching and researching in the fields of Management and Economics at different universities located in the UK. For the Vietnamese lecturers, they were teaching some modules at both undergraduate and postgraduate levels, and importantly they gained substantial experiences conducting research in these two fields. For the Vietnamese doctoral researchers, their research expertise was also related to the objectives of the questionnaire. For each item, these 15 participants were asked to specifically give a score ranging from 1 to 5 (1 – highly irrelevant, 2 – moderately irrelevant, 3 – neutral, 4 – moderately relevant, and 5 – highly relevant). Their ratings were used to examine three content validity indices including Lawshe's content validity ratio (CVR), Aiken's V coefficient and Penfield's interval scores.

The CVR (Lawshe, 1975) is defined as the degree to which the panel of experts (i.e. 15 lecturers and researchers in this study) specifies the necessity of an item. The formula for computation of CVR is presented as follow:

$$CVR=\;\frac{n_{e}-\frac{N}{2}}{N:2}$$

where n_e_ represents the number of experts rating the item is essential (i.e. highly relevant or moderately relevant) and N means the total number of experts (Lawshe, 1975). Given that there were 15 participants (N = 15), the cut-off value of CVR in this study was estimated at 0.15 (at p = 0.05). As indicated in Table 2, all of the questionnaire items in the five constructs (i.e. CS, EI, OSE, SS, and SP) were higher than the cut-off value.

**Table 2:** Content validity through Lawshe's CVR.

Item	e (indicating the relevance of the item)	CVR by item
CS1	12	0.600
CS2	10	0.333
CS3	11	0.467
CS4	12	0.600
CS5	12	0.600
CS6	12	0.600
CS7	12	0.600
CS8	12	0.600
CS9	12	0.600
EI1	13	0.733
EI2	11	0.467
EI3	10	0.333
EI4	10	0.333
EI5	9	0.200
EI6	11	0.467
EI7	11	0.467
EI8	12	0.600
EI9	12	0.600
EI10	11	0.467
OSE1	10	0.333
OSE2	10	0.333
OSE3	9	0.200
OSE4	11	0.467
OSE5	12	0.600
OSE6	12	0.600
OSE7	12	0.600
SS1	11	0.467
SS2	14	0.867
SS3	11	0.467
SS4	9	0.200
SS5	12	0.600
SS6	12	0.600
SS7	10	0.333
SS8	11	0.467
SP1	12	0.600
SP2	12	0.600
SP3	12	0.600
SP4	12	0.600
SP5	12	0.600

The second index of content validity is the content relevance. In order to ensure that the items reach the content relevance, Aiken's content validity coefficient or Aiken's V (Aiken, 1985) and confidential interval (CI) (Penfield, 2003) were taken into consideration. The formula for computation of Aiken's V is presented as follow:

$$V=\frac{S}{n*\left(c-1\right)}$$

where S represents the scores given by each rater minus the lowest score in the rating range (normally, this is 1 in the 5-point rating scale); n represents the number of raters in the study; and c represents the number of options that the raters can choose from. V ranges from 0 to 1.0, which means that a higher value demonstrates higher content validity.

Nevertheless, the results obtained from Aiken's V are not sufficient; the content relevance should be further calculated with CI (Penfield, 2003; Penfield and Giacobbi, 2004). Following the results of Aiken's V, the formulas for computation of the lower (L) and upper (U) limits are presented as follow:

$$L=\;\frac{2nKV+z^{2}-z\sqrt{4nKV\;\left(1-V\right)+z^{2}}}{2\;\left(nK+z^{2}\right)}$$

$$U=\;\frac{2nKV+z^{2}+\;z\sqrt{4nKV\;\left(1-V\right)+z^{2}}}{2\;\left(nK+z^{2}\right)}$$

where n represents the number of raters in the study; K means the highest point minus the lowest point in the rating range (normally, they are 1 and 5 showing highly irrelevance and highly relevance, respectively); and z represents the confidence value (z_90%_ = 1.65; z_95%_ = 1.96).

The results of Aiken's V and CI are presented in Table 3, showing that all the questionnaire items satisfied the content validity. More specifically, the coefficients ranging from 0.593 to 0.964 were within the range of 0.20.

Step four: Pilot study

**Table 3:** Results of ratings, values of Aiken's V and score confidence interval (CI).

Item	Rating frequency						90% CI			95% CI		
	5	4	3	2	1	V	Lower limit	Upper limit	Typical length	Lower limit	Upper limit	Typical length
CS1	10	2	3	0	0	0.867	0.779	0.924	0.145	0.759	0.931	0.172
CS2	7	3	4	1	0	0.767	0.667	0.844	0.178	0.646	0.856	0.210
CS3	8	3	4	0	0	0.812	0.716	0.881	0.165	0.696	0.891	0.195
CS4	12	0	3	0	0	0.9	0.818	0.948	0.130	0.799	0.953	0.155
CS5	11	1	3	0	0	0.883	0.797	0.935	0.138	0.778	0.942	0.164
CS6	12	0	2	1	0	0.883	0.797	0.935	0.138	0.778	0.942	0.164
CS7	9	3	3	0	0	0.85	0.759	0.911	0.152	0.739	0.919	0.180
CS8	8	4	3	0	0	0.833	0.740	0.898	0.158	0.719	0.907	0.187
CS9	9	3	3	0	0	0.85	0.759	0.911	0.152	0.739	0.919	0.180
EI1	12	1	2	0	0	0.917	0.839	0.959	0.121	0.820	0.964	0.144
EI2	10	1	3	1	0	0.833	0.740	0.898	0.158	0.719	0.907	0.187
EI3	10	0	5	0	0	0.833	0.740	0.898	0.158	0.719	0.907	0.187
EI4	7	3	5	0	0	0.783	0.684	0.857	0.174	0.663	0.869	0.205
EI5	5	4	6	0	0	0.733	0.630	0.816	0.185	0.610	0.828	0.219
EI6	9	2	4	0	0	0.833	0.740	0.898	0.158	0.719	0.907	0.187
EI7	10	1	4	0	0	0.85	0.759	0.911	0.152	0.739	0.919	0.180
EI8	11	1	3	0	0	0.883	0.797	0.935	0.138	0.778	0.942	0.164
EI9	9	3	3	0	0	0.850	0.759	0.911	0.152	0.739	0.919	0.180
EI10	11	0	4	0	0	0.867	0.779	0.924	0.145	0.759	0.931	0.172
OSE1	7	3	5	0	0	0.783	0.684	0.857	0.174	0.663	0.869	0.205
OSE2	8	2	5	0	0	0.800	0.703	0.871	0.169	0.682	0.882	0.200
OSE3	7	2	5	1	0	0.750	0.648	0.830	0.182	0.628	0.842	0.215
OSE4	10	1	4	0	0	0.850	0.759	0.911	0.152	0.739	0.919	0.180
OSE5	10	2	3	0	0	0.867	0.779	0.924	0.145	0.759	0.931	0.172
OSE6	11	1	3	0	0	0.883	0.797	0.935	0.138	0.778	0.942	0.164
OSE7	11	1	3	0	0	0.883	0.797	0.935	0.138	0.778	0.942	0.164
SS1	9	2	4	0	0	0.833	0.740	0.898	0.158	0.719	0.907	0.187
SS2	9	5	1	0	0	0.883	0.797	0.935	0.138	0.778	0.942	0.164
SS3	11	0	4	0	0	0.867	0.779	0.924	0.145	0.759	0.931	0.172
SS4	5	4	5	1	0	0.717	0.613	0.802	0.189	0.593	0.815	0.223
SS5	12	0	3	0	0	0.900	0.818	0.948	0.130	0.799	0.953	0.155
SS6	11	1	3	0	0	0.883	0.797	0.935	0.138	0.778	0.942	0.164
SS7	7	3	5	0	0	0.783	0.684	0.857	0.174	0.663	0.869	0.205
SS8	10	1	4	0	0	0.850	0.759	0.911	0.152	0.739	0.919	0.180
SP1	11	1	3	0	0	0.883	0.797	0.935	0.138	0.778	0.942	0.164
SP2	10	2	3	0	0	0.867	0.779	0.924	0.145	0.759	0.931	0.172
SP3	8	4	3	0	0	0.833	0.740	0.898	0.158	0.719	0.907	0.187
SP4	11	1	3	0	0	0.883	0.797	0.935	0.138	0.778	0.942	0.164
SP5	10	2	3	0	0	0.867	0.779	0.924	0.145	0.759	0.931	0.172

Based on the refined survey from the Delphi studies, a pilot study was conducted with a sample of 50 participants (7 male and 43 female). In terms of age, they mainly came from two groups: 21–25 (12 participants) and 26–30 (18 participants). This enables us to observe patterns in participants’ answers and any issues with the questionnaire survey in order to ensure the quality of content and reliability of measures. The results show that the internal consistency reliability was excellent, indicating that the questionnaire was appropriate for the main study.

Step five: Main study

After completing this pilot study, we proceed to finalize the questionnaire survey and send out to randomly consumers via online channels including Facebook. The target participants are Vietnamese people who may have interests or experience in shopping online products like beauty and cosmetics. A total of 334 participants took part in the instrument validation phase of the study. The sample was predominantly female (69.25%). Detailed demographic data are presented in Table 4.

**Table 4:** Demographic information of respondents.

	Male (n_male_ = 76)	Female (n_female_ = 259)	Total (N = 334)
Age range			
18–20	8 (10.5%)	16 (6.6%)	24 (7.5%)
21–25	15 (19.7%)	55 (21.2%)	70 (20.9%)
26–30	22 (28.9%)	62 (23.9%)	84 (25.1%)
31–35	19 (25.0%)	84 (32.4%)	103 (30.7%)
Over 35	12 (15.8%)	41 (15.8%)	53 (15.8%)
Marital status			
Single	30 (39.5%0	84 (32.8%)	114 (34.3%)
Married	21 (27.6%)	94 (36.3%)	115 (34.3%)
Separated	6 (7.9%)	31 (12.0%)	37 (11.0%)
Divorce	19 (25.0%)	49 (18.9%)	68 (20.2%)
Highest qualifications			
High School Diploma	9 (11.8%)	26 (10.4%)	35 (10.7%)
Bachelor's Degree	28 (36.8%)	71 (27.4%)	99 (29.6%)
Masters' Degree	33 (43.4%)	136 (52.5%)	169 (50.3%)
Doctoral Degree	6 (7.9%)	25 (9.7%)	31 (9.3%)
Salary			
<8 million VND	10 (13.2%)	30 (12.0%)	40 (12.2%)
8–15 million VND	10 (13.2%)	67 (25.9%)	77 (22.9%)
15- 25 million VND	20 (26.3%)	82 (31.7%)	102 (30.4%)
25- 35 million VND	13 (17.1%)	51 (19.7%)	64 (19.0%)
>35 million VND	23 (30.3%)	28 (10.8%)	51 (15.2%)

Based on 334 full responses have been received, we use Exploratory Factor Analysis (EFA) to test for 167 observations to ensure that all items in each scale reflected sufficiently the scope of each construct. In other words, this technique helps to identify the underlying dimensions of consumer satisfaction. Furthermore, we also employ Confirmatory Factor Analysis (CFA) to validate the constructs and items in questionnaire. To do this, we have used the sample of 167 responses.

The EFA and CFA were recommended to be performed in independent samples (Knekta et al., 2019), particularly the EFA was undertaken with half of the sample and the CFA was followed with the other half of the sample. This is because the results from the EFA should be confirmed with the CFA on different sample; otherwise the CFA will repeat the relationships derived from the EFA results.

## 4 Validating the five-construct model of CS and questionnaire

### 4.1 Exploratory factor analysis (EFA)

Regarding survey items on CS questionnaire, all 39 items were run through and validated by using principal component analysis (PCA) procedure, followed by the varimax rotation method. The factorability of these items was examined by using Kaiser-Meyer-Olkin (KMO) measure of sampling adequacy test and Bartlett's test of sphericity in order to determine whether the sample size was sufficient, and the data were suitable for factor analysis. As a result, the validity of the PCA was confirmed by a satisfactory value of the KMO test for sampling adequacy, KMO = 0.827, exceeding the 0.60 suggested limit, and the statistically significant Bartlett's test of sphericity (χ2 (741) = 3221.178, p = 0.000). These measures affirmed that the data were satisfactory for the EFA procedure and favourable for explaining CS (Khan and Adil, 2013), revealing five extracted factors having an eigenvalue greater than 1 and accounting for 52.014% of the total variance. In addition, based on the decision for EFA (cut-off limit = 0.50), two items were excluded from the analyses and further analyses were conducted with the remaining 37 items (Table 5).

**Table 5:** Rotated factor loadings for the five factors (N = 167, total variance explained is 52.014%, α = 0.899, thirty-seven items).

	Items	Loading
**Factor 1: Customer satisfaction**		
CS5	I will make more purchases for beauty and cosmetics online products in the future (*Tôi sẽ mua nhiều sản phẩm làm đẹp và mỹ phẩm trực tuyến trong tương lai*).	*0.830*
CS2	The online beauty and cosmetics shop always meets my needs (*Cửa hàng mỹ phẩm và làm đẹp trực tuyến luôn đáp ứng nhu cầu của tôi*).	*0.822*
CS9	Overall, I encourage friends and others to purchase goods from online beauty and cosmetics shops (*Nhìn chung, tôi khuyến khích bạn bè và những người khác mua hàng hóa từ các cửa hàng mỹ phẩm và làm đẹp trực tuyến*).	*0.811*
CS4	Overall, I say positive things about beauty and cosmetics online shops to other people (*Nhìn chung, tôi sẽ nói những điều tích cực về các cửa hàng trực tuyến làm đẹp và mỹ phẩm cho người khác*).	*0.805*
CS6	The online beauty and cosmetics shops are getting close to the ideal online retailer (*Cửa hàng mỹ phẩm và làm đẹp trực tuyến đang dần trở thành những nhà bán lẻ trực tuyến lý tưởng*).	*0.801*
CS3	Overall, if problems arise, one can expect to be treated fairly by online beauty and cosmetics shops (*Nhìn chung, nếu có vấn đề phát sinh, người ta có thể mong đợi được đối xử công bằng bởi các cửa hàng mỹ phẩm và làm đẹp trực tuyến*).	*0.797*
CS1	The overall quality of the beauty and cosmetics online products is excellent (*Chất lượng tổng thể của dịch vụ cho sản phẩm làm đẹp và mỹ phẩm trực tuyến là tuyệt vời*).	*0.758*
CS7	Overall, I recommend beauty and cosmetics shops to anyone who seeks my advice (*Nhìn chung, tôi giới thiệu các cửa hàng làm đẹp và mỹ phẩm cho bất cứ ai tìm kiếm lời khuyên của tôi*).	*0.711*
CS8	Overall, online beauty and cosmetics shops are genuinely interested in customer's welfare (*Nhìn chung, các cửa hàng mỹ phẩm và làm đẹp trực tuyến thực sự quan tâm đến phúc lợi của khách hàng*).	*0.700*
**Factor 2: External Incentives**		
EI6	More product specials or promotions (of beauty and cosmetics products) (*Nhiều sản phẩm đặc biệt hoặc chương trình khuyến mãi (của các sản phẩm làm đẹp và mỹ phẩm)*).	*0.702*
EI8	More products with free shipping (of beauty and cosmetics products) (*Nhiều sản phẩm được miễn phí vận chuyển (của các sản phẩm làm đẹp và mỹ phẩm)*).	*0.694*
EI9	Diversification of products (of beauty and cosmetics products) (*Đa dạng hóa sản phẩm (sản phẩm làm đẹp và mỹ phẩm)*).	*0.694*
EI7	Detailed product specifications and features (of beauty and cosmetics products) (*Thông số kỹ thuật và tính năng của sản phẩm được trình bày rất chi tiết và dễ hiểu (của sản phẩm làm đẹp và mỹ phẩm)*).	*0.676*
EI4	Wide diversification of advertising (of beauty and cosmetics products) (*Đa dạng hóa quảng cáo (của các sản phẩm làm đẹp và mỹ phẩm)*).	*0.644*
EI1	Product only available online (of beauty and cosmetics products) (*Sản phẩm chỉ có sẵn khi mua trực tuyến (của các sản phẩm làm đẹp và mỹ phẩm)*).	*0.613*
EI5	Product is purchased according to the notability of brands (of beauty and cosmetics products) (*Sản phẩm được mua theo sự danh giá của các thương hiệu (của các sản phẩm làm đẹp và mỹ phẩm)*).	*0.606*
EI2	Provide special product bundles (of beauty and cosmetics products) (*Cung cấp các gói sản phẩm đặc biệt (của các sản phẩm làm đẹp và mỹ phẩm)*).	*0.599*
EI3	Price cheaper than physical stores (of beauty and cosmetics products) (*Giá rẻ hơn so với các cửa hàng hiện hữu (của các sản phẩm làm đẹp và mỹ phẩm)*).	*0.587*
EI10	Product not easy to buy on the market (of beauty and cosmetics products) (*Có thể mua được sản phẩm không dễ mua trên thị trường (của các sản phẩm làm đẹp và mỹ phẩm)*).	*0.528*
**Factor 3: Online Shopping Experience**		
OSE2	I am satisfied with most recently purchased product (of any type of products) (*Tôi hài lòng với sản phẩm được mua gần đây nhất khi mua hàng trực tuyến (của bất kỳ các loại sản phẩm)*).	*0.812*
OSE3	I care about brand reputation of beauty and cosmetics (*Tôi quan tâm đến uy tín thương hiệu của các sản phẩm làm đẹp và mỹ phẩm khi mua hàng trực tuyến*).	*0.750*
OSE1	I am satisfied with mostly recently purchased beauty and cosmetics online (*Tôi hài lòng với hầu hết các sản phẩm làm đẹp và mỹ phẩm được mua trực tuyến gần đây*).	*0.729*
OSE4	I care about attractive packaging of online beauty and cosmetics products (*Tôi quan tâm đến sự hấp dẫn của thiết kế bao bì của các sản phẩm làm đẹp và mỹ phẩm khi mua hàng trực tuyến*).	*0.715*
OSE5	When shopping beauty and cosmetics products online, I care about professionalism of service personnel (*Khi mua hàng trực tuyến, tôi ưu tiên các sản phẩm làm đẹp và mỹ phẩm nếu nhân viên tư vấn và phục vụ chuyên nghiệp*).	*0.535*
OSE7	I care about natural ingredients of online beauty and cosmetics products (*Tôi quan tâm đến sản phẩm làm đẹp và mỹ phẩm có nhiều thành phần tự nhiên khi mua hàng trực tuyến*).	*0.517*
OSE6	I was recommended to purchase beauty and cosmetics products by experts such as doctors and beauty vloggers (*Tôi được khuyên nên mua sản phẩm làm đẹp và mỹ phẩm trực tuyến bởi các chuyên gia (beauty vloggers, bác sĩ chuyên khoa, ...)*).	*0.512*
**Factor 4: Seller Services**		
SS1	There is a convenient return or replacement process (of beauty and cosmetics products) (*Có sự thuận tiện trong quá trình hoàn trả hoặc thay đổi (của các sản phẩm làm đẹp và mỹ phẩm) khi mua hàng trực tuyến*).	*0.753*
SS2	There is diversification of payment methods (of beauty and cosmetics products) (*Có sự đa dạng hóa các phương thức thanh toán (của các sản phẩm làm đẹp và mỹ phẩm)*).	*0.614*
SS5	There has a fast response to questions made by customer (of beauty and cosmetics products) (*Có phản hồi nhanh chóng cho các câu hỏi của khách hàng khi mua hàng trực tuyến (về các sản phẩm làm đẹp và mỹ phẩm)*).	*0.592*
SS7	The website has no difficulties with making a payment online (of beauty and cosmetics products) (*Trang web không gặp khó khăn gì khi thanh toán trực tuyến (các sản phẩm làm đẹp và mỹ phẩm)*).	*0.569*
SS6	There is a fast delivery (of beauty and cosmetics products) (*Giao hàng nhanh chóng (các sản phẩm làm đẹp và mỹ phẩm)*).	*0.547*
SS3	There is diversification of delivery methods (of beauty and cosmetics products) (*Có sự đa dạng hóa các phương thức giao hàng (của các sản phẩm làm đẹp và mỹ phẩm*)).	*0.534*
SS4	The information on the website is pretty much what I need to carry out my tasks (of beauty and cosmetics products) (*Thông tin trên trang web là khá tốt, đáp ứng được những gì tôi cần để thực hiện các nhu cầu của mình (về các sản phẩm làm đẹp và mỹ phẩm)*).	*0.527*
SS8	The product was not damaged during delivery (of beauty and cosmetics products) (*Sản phẩm không bị hư hỏng trong quá trình giao hàng (của các sản phẩm làm đẹp và mỹ phẩm)*).	*0.508*
**Factor 5: Security and Privacy**		
SP3	I trust the website administrators will not misuse my personal information (of beauty and cosmetics products) (*Tôi tin rằng các quản trị viên trang web sẽ không lạm dụng thông tin cá nhân của tôi (về các sản phẩm làm đẹp và mỹ phẩm)*).	*0.755*
SP1	I feel safe in my transactions with the online shop (of beauty and cosmetics products) (*Tôi cảm thấy an toàn trong các giao dịch của mình với cửa hàng trực tuyến (các sản phẩm làm đẹp và mỹ phẩm)*).	*0.719*
SP2	I trust the online shop to keep my personal information safe (of beauty and cosmetics products) (*Tôi tin tưởng các cửa hàng trực tuyến đã giữ thông tin cá nhân của tôi an toàn (của các sản phẩm làm đẹp và mỹ phẩm)*).	*0.682*

The first factor labelled ‘Customer satisfaction’ (CS) involved nine items that captured 21.920% of the variance. Factor two was termed ‘External incentives’ (EI) that included ten items and explained 13.608% of the variance. Seven items were extracted and loaded onto factor three named ‘Online shopping experience’ (OSE) with 6.869% of the total variance. The fourth factor labelled ‘Seller service’ (SS) included eight items accounting for 5.377% of the total variance. The fifth factor named ‘Security and Privacy’ (SP) included six items and captured 2.602% of the variance.

Internal consistency reliability was tested by using Cronbach's Alpha coefficients (α) for the five explored constructs. SS (α = 0.759) and SP (α = 0.717) were greater than 0.70, showing acceptable consistency. EI (α = 0.866) and OSE (α = 0.840) were greater than 0.80 and showed good consistency, while CS (α = 0.931) was greater than 0.90 and displayed excellent consistency (Henson, 2001). These results revealed that the five emerged factors were all reliable for examining CS.

### 4.2 Confirmatory factor analysis (CFA)

Next, the CFA, which aims to test the construct validity and reliability of the survey, was conducted with the second sample of 167 respondents. Hair et al. (2013) suggest that the factor loading, t-values, average variance extracted (AVE) and composite reliability (CR) were appropriate for assessing the construct validity and reliability. Previous studies on CFA indicate that no perfect index for assessing model goodness of fit exists. Hence, we consider using variety of approaches such as Minimum Discrepancy per Degree of freedom (CMIN/DF), Goodness-of-fit index (GFI), Tucker Lewis Index (TLI), Comparative Fit Index (CFI), and Root Mean Square Error of Approximation (RMSEA). However, it has been recommended that the GFI should be avoided when deciding what indices to report (Sharma et al., 2005). This is because the GFI is sensitive and lacks sophistication to become a stand-alone index (Hooper et al., 2008).

We employ convergent and discriminant validity for the purpose of examining the construct validity. The former refers to the degree where the variables in a particular dimension indicate the same construct. The latter is the degree to which the dimensions identified are independent. Results of the CFA confirmed the good model fit for a five-factor model measuring CS.

#### 4.2.1 Convergent validity

**Figure 5:**
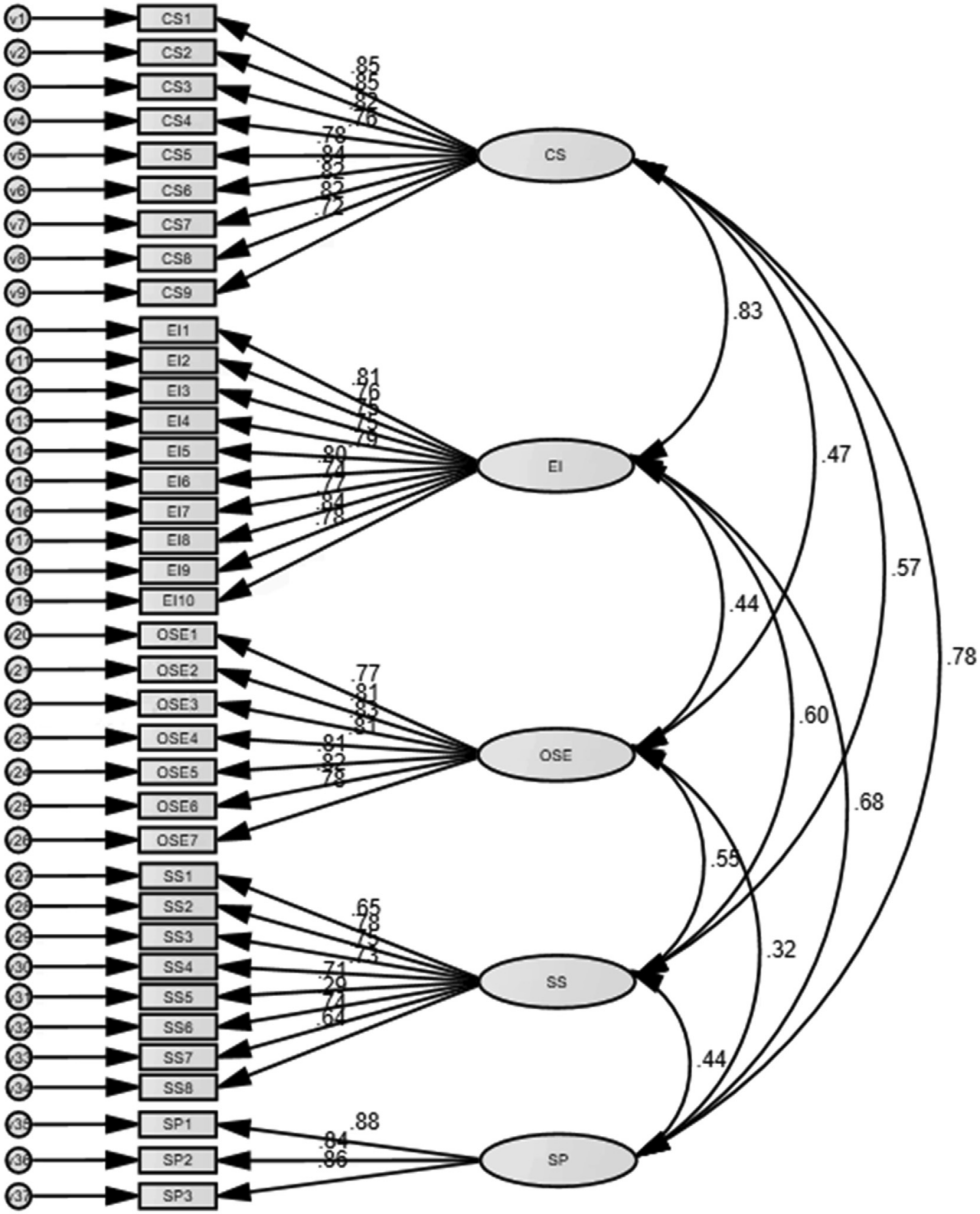
CFA result.

The summary of the accepted and observed values for the fit indices is present in Table 6 and Figure 5. The overall model fit indices showed that goodness-of-fit measure was within the acceptable range (χ2 _(619)_ = 1015.949, p = 0.000, CMIN/DF = 1.641, IFI = 0.914, TLI = 0.907, CFI = 0.913, and RMSEA = 0.062). In addition, Table 6 shows that all values of construct reliability (CR) surpassed the acceptable level of 0.70 (Raykov, 1997), and all estimates of average variance extracted (AVE) were higher than 0.50 (Nunnally and Bernstein, 1994). These results supported a clear convergent validity for all constructs.

**Table 6:** Results of convergent validity.

Construct	M	SD	AVE	CR
CS	3.587	0.798	0.554	0.879
EI	3.589	0.813	0.557	0.895
OSE	3.359	1.126	0.684	0.898
SS	3.646	0.728	0.537	0.852
SP	3.340	0.985	0.555	0.661

Note. M = Mean; SD = Standard deviation; AVE = Average variance explained; CR = Composite reliability.

#### 4.2.2 Discriminant validity

Discriminant validity focuses on whether the correlations between items measuring different construct are relatively low. Correlations of more than 0.80 imply an overlap between the constructs and poor discriminant validity (Brown, 2014). As depicted in Table 7, the results showed that the highest correlation was 0.772, thereby satisfying the conditions of discriminant validity.

**Table 7:** Correlations between five subscales of the questionnaire (N = 167).

Variable	1	2	3	4	5
1. CS	1.0				
2. EI	0.772∗∗∗	1.0			
3. OSE	0.449∗∗	0.417∗∗∗	1.0		
4. SS	0.526∗∗∗	0.544∗∗∗	0.489∗∗∗	1.0	
5. SP	0.728∗∗∗	0.625∗∗∗	0.293∗∗∗	0.417∗∗∗	1.0

Note. ∗p < 0.1, ∗∗p < 0.05, ∗∗∗p < 0.01.

### 4.3 Evaluation of the structural model: hypothesis testing

**Figure 6:**
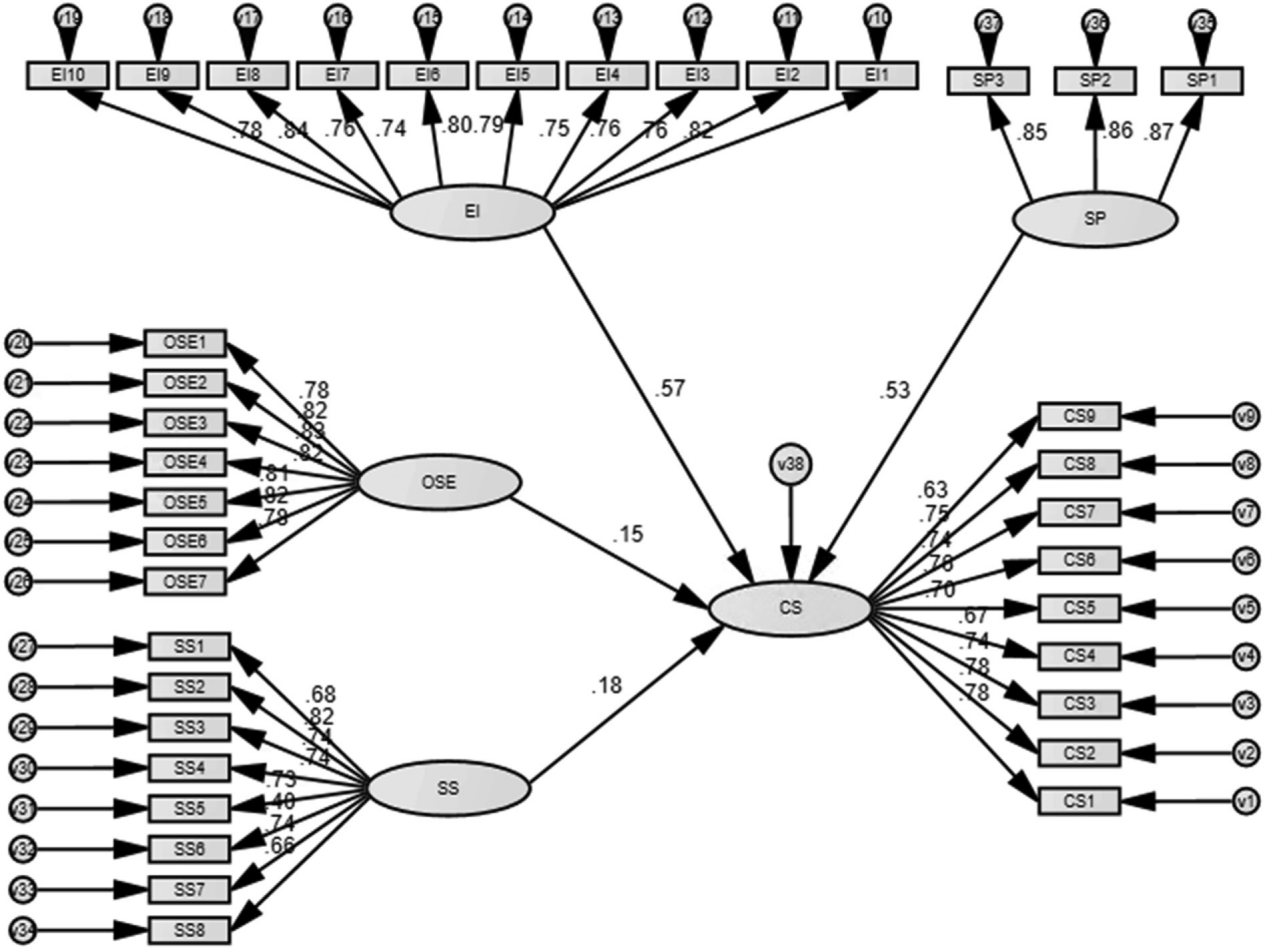
SEM result.

The overall model fit indices showed that goodness-of-fit measure was within the acceptable range (χ2 _(625)_ = 1255.418, p = 0.000, CMIN/DF = 2.009, IFI = 0.910, TLI = 0.902, CFI = 0.908, and RMSEA = 0.078). In addition, p-value of each pair of constructs are lower than 5% or 1%, which suggests a positive effect of OSE on CS, that of SS on CS, that of EI on CS and that of SP on CS. (See Table 8 and Figure 6). These results are consistent with all our hypotheses and previous literature. The R-squared is 0.66, suggesting that the four constructs have explained 66% of the variance of CS.

**Table 8:** Results of the structural equation modelling.

	Casual path	Path coefficient	p value	Supported?
H_1_	OSE→ CS	0.15∗∗	0.012	**Yes**
H_2_	SS → CS	0.18∗∗∗	0.004	**Yes**
H_3_	EI → CS	0.57∗∗	0.000	**Yes**
H_4_	SP → CS	0.53∗∗∗	0.000	**Yes**

Note. ∗p < 0.1, ∗∗p < 0.05, ∗∗∗p < 0.01.

## 5 Discussions and conclusions

This study develops a five-construct theoretical model of CS with beauty and cosmetics online businesses and validates the questionnaire created from that model. 334 responses have been received and they are relatively equally used for EFA and CFA technique which helps to prove the reliability and validity of our survey and in turn, our theoretical model. Compared to prior CS research on cosmetics, we shed a light on the aspects of customers’ experiences with their purchasing of beauty and cosmetics in online shopping that have notable effects on their satisfaction with these services.

As stated in the result section, internal consistency reliability has been proved through using Cronbach's Alpha coefficients (α) for the five explored constructs. These results imply that the five emerged factors were all reliable for examining CS. Furthermore, after EFA tests, we proceed to employ the CFA technique to investigate the construct reliability and validity of the questionnaire. In order to test the construct validity of the instrument, both convergent and discriminant validity were computed, and the results all support a clear convergent validity for all constructs and satisfy the conditions of discriminant validity.

Finally, we use SEM model to test the hypotheses set and find that all hypotheses are supported. This suggests that constructs of our model including OSE, SS, EI and SP are important factors affecting CS of online businesses in beauty and cosmetics sector. This is in line with previous literature and our theoretical model. Taken together, the current study contributes to providing a reliable and valid questionnaire which fully reflect for our constructed theoretical model of five constructs including Online shopping experience; External incentives; Customer service; and Security/privacy; and personal characteristics. Our second study will use these results to examine which constructs significantly affecting the CS in the same sector and same online businesses in Vietnam.

Our study has several important implications. First, our five factor model can be used as a baseline of future studies in the same field. Second, our findings are important to both existing online businesses and new/future entrants to the sector; for example, they can consider our factors to establish strategies and plans to enhance the customer satisfaction and ultimately their revenue and profits. Third, our study is the first to develop and validate a comprehensive questionnaire in Vietnamese, so future studies in the same market can use this as their reference and do not need to re-validate it. Likewise, online businesses in the beauty and cosmetics sector can also use our questionnaire survey as a starting point for their marketing survey, then modify it to fit well the context of their business.

One of our limitations is that the number of male and female participants are not equal. In addition, some of other factors related to psychology are missing from the model. We therefore call for future research to add psychological factors into their study to exploit how these variables can influence their overall satisfaction when purchasing online beauty and cosmetics products. Furthermore, because our research is only conducted for Vietnamese market and beaty and cosmetics sector, therefore, its’ findings may be not generalised for other countries and other industries. Further studies, hence, can expand our results by testing for other markets and sectors.

## Declarations

### Author contribution statement

T.T.N. Nguyen: Conceived and designed the experiments; Performed the experiments; Analyzed and interpreted the data; Contributed reagents, materials, analysis tools or data; Wrote the paper.

### Funding statement

This research did not receive any specific grant from funding agencies in the public, commercial, or not-for-profit sectors.

### Competing interest statement

The authors declare no conflict of interest.

### Additional information

No additional information is available for this paper.
